# Exploring Sensitive Label-Free Multiplex Analysis with Raman-Coded Microbeads and SERS-Coded Reporters

**DOI:** 10.3390/bios12020121

**Published:** 2022-02-16

**Authors:** Umar Azhar, Qazi Ahmed, Saira Ishaq, Zeyad T. Alwahabi, Sheng Dai

**Affiliations:** 1 School of Chemical Engineering and Advanced Materials, The University of Adelaide, Adelaide, SA 5005, Australia; umar_azhar@hotmail.com (U.A.); engrqaziahmed@hotmail.com (Q.A.); saira_chem@yahoo.com (S.I.); 2 School of Chemical and Process Engineering, University of Leeds, Leeds LS2 9JT, UK

**Keywords:** SERS, Raman, microbeads, multiplex, immunoassays

## Abstract

Suspension microsphere immunoassays are rapidly gaining attention in multiplex bioassays. Accurate detection of multiple analytes from a single measurement is critical in modern bioanalysis, which always requires complex encoding systems. In this study, a novel bioassay with Raman-coded antibody supports (polymer microbeads with different Raman signatures) and surface-enhanced Raman scattering (SERS)-coded nanotags (organic thiols on a gold nanoparticle surface with different SERS signatures) was developed as a model fluorescent, label-free, bead-based multiplex immunoassay system. The developed homogeneous immunoassays included two surface-functionalized monodisperse Raman-coded microbeads of polystyrene and poly(4-tert-butylstyrene) as the immune solid supports, and two epitope modified nanotags (self-assembled 4-mercaptobenzoic acid or 3-mercaptopropionic acid on gold nanoparticles) as the SERS-coded reporters. Such multiplex Raman/SERS-based microsphere immunoassays could selectively identify specific paratope–epitope interactions from one mixture sample solution under a single laser illumination, and thus hold great promise in future suspension multiplex analysis for diverse biomedical applications.

## 1. Introduction

Nowadays, accurate diagnosis of human malignant diseases at the earliest stage is vital to curtail the side effects of surgical procedures and radio- and/or chemotherapies, subsequently followed by costly treatment. Typically, infectious or immune-system-related diseases generate various antigens as the first sign of abnormality in organisms. The most representative method for analysis of such disease biomarkers is immunoassays, realized by the immune recognition between antigens and relevant specific antibodies. In clinical applications, diseases as complex as cancers always necessitate multiplex diagnostics; that is, to simultaneously analyze multiple analytes from a single sample measurement. Hence, high sensitivity and high throughput are the desired features in developing novel multiplex immunoassays.

As per signal readout arrangements, immunoassays can be separated into a few classifications, including the enzyme-linked immunosorbent assay (ELISA) [1,2,3], luminescence [4,5], colorimetric [6,7], fluorescence [8,9], surface plasmon resonance (SPR) [10,11], and surface-enhanced Raman scattering (SERS) [12,13]. To date, traditional biological detection strategies such as ELISA and fluorescence are widely used in various clinical applications, but suffer from various shortcomings, such as high background noise, high spectral overlap, low sensitivity, and photobleaching. Among these readout technologies, SERS-based immunoassays have realized significant application in multiplex analyses. SERS has shown great promise in biomolecular analysis, as it only requires a small amount of body fluids (e.g., blood, urine, ascites, or saliva) for repetitive and timely examination, rather than complicated tissue analysis. In addition, SERS-based immunoassays achieve a low limit of detection (LOD), large dynamic range, and high sensitivity due to the enhanced Raman signals offered by SERS-active molecules physically adsorbed on noble metal nanoparticle surfaces [14,15,16]. In addition, the full width at half wavelength (FWHW) of a Raman signal is much narrower than that of fluorescence. Therefore, the interference caused by overlapping broad emission spectra will be greatly decreased between different SERS coding elements, which makes it an efficient encoding system for multiplex analysis.

To date, substrate-based SERS immunoassays have been widely utilized in biochemical and biomedical applications for the identification of different biological targets; for example, proteins [17,18,19], nucleic acids [20,21], infection [22], cells [23,24], and poisons [25]. This type of biomolecular recognition uses substrate-immobilized, immuno-functionalized SERS nanoprobes to recognize various target analytes. In a typical encrypting method, distinct Raman signatures of nanoprobes can be obtained by selecting different SERS-active molecules or their combinations [26,27]. As such, a series of SERS-coded tags have been developed for substrate-based analysis [28,29,30,31,32,33,34,35,36,37]. Although this technique provides high-sensitivity multiplex analysis, it is limited by high throughput and a large dynamic detection range. On the other hand, bead-based immunoassays have gained exponential recognition in recent years in developing novel SERS-based multiplex bioanalysis platforms. Compared to these well-established bead-based fluorescence immunoassays, the bead-based Raman and/or SERS immunoassays show obvious advantages such as label-free, narrow vibration spectra; high sensitivity; and a single excitation wavelength, in turn offering higher multiplexing capability. In particular, these Raman beads not only act as a platform for immune bead support, but also as a label-free code [38,39,40].

**Figure 1:**
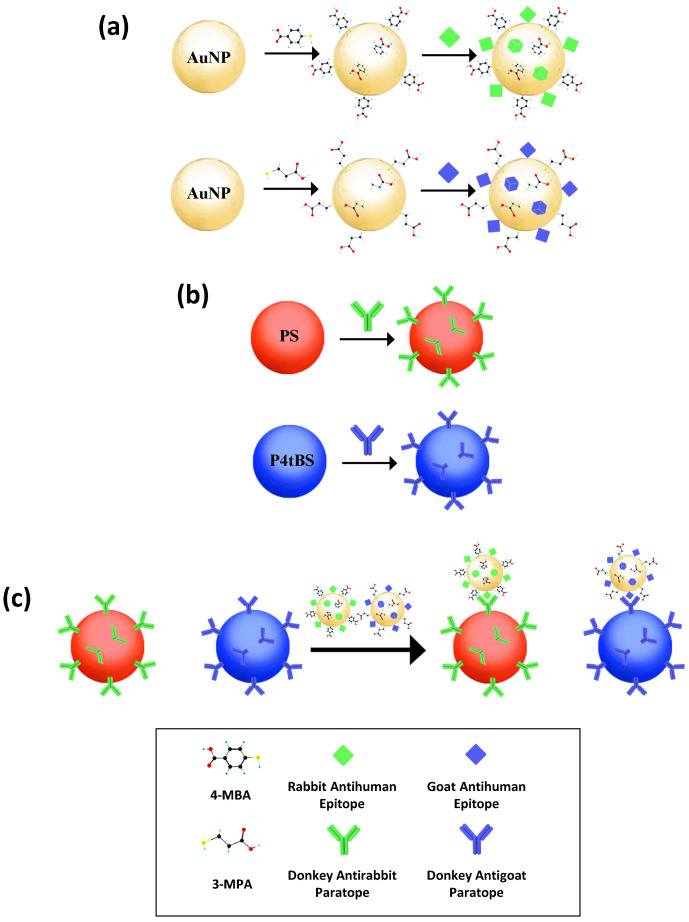
Schematic description of the preparation of: (**a**) SERS-coded reporters; (**b**) surface functionalized Raman-coded polymeric microbeads; (**c**) the resulting bead-based multiplex immunoassays.

In this study, we developed a model bead-based multiplex immunoassay system in the presence of Raman-coded microbeads and SERS-coded nanotags for simultaneous multiplex analysis of epitopes (antigens). As illustrated in Figure 1, the SERS-coded nanotags were simply prepared by the self-assembly of functional SERS-active molecules to gold nanoparticle (AuNP) surfaces, and the SERS-coded reporters obtained from interaction of SERS-coded nanotags to different epitopes. On the other hand, paratopes (antibodies) could be immobilized on the surface of Raman-coded microbeads. In an immunocomplex mixture of various functionalized SERS reporters and Raman microbeads, Raman imaging from one sample measurement allowed us to accurately identify analyte interactions by tailoring different vibrational bands of the Raman/SERS signatures of microbeads and nanotags. Our results showed the abilities of applying Raman spectroscopy and imaging in bead-based multiplex bioanalysis with high sensitivity and high specificity. This holds great promise for future applications in multiplexing, high-throughput screening, and detection of complex human diseases.

## 2. Materials and Methods

### 2.1. Materials

Gold chloride trihydrate (HAuCl_4_·3H_2_O), 4-mercaptobenzoic acid (4-MBA), 3-mercaptopropionic acid (3-MPA), styrene, 4-tert-butylstyrene, polyvinylpyrrolidone (PVP 360,000) and bovine serum albumin (BSA) were procured from Sigma-Aldrich (St. Louis, MO, USA). Acrylic acid (AA), 2,2′-Azobis(2-methylpropionitrile) (AIBN), *N*-(3-dimethylaminopropyl)-*N*′-ethylcarbodiimide hydrochloride (EDC), and *N*-hydroxysuccinimide (NHS) were procured from Acros Organics (Geel, Belgium). Tri-sodium citrate dehydrate was attained from Prolabo (Phnom Penh, Cambodia). AffiniPure donkey antirabbit IgG (paratope in this study), AffiniPure donkey antigoat IgG (paratope in this study), AffiniPure rabbit antihuman IgG (epitope in this study), and AffiniPure goat antihuman IgG (epitope in this study) were obtained from Jackson ImmunoResearch (West Grove, PA, USA). These lyophilized IgG samples (H + L) were polyclonal in sterile filtered liquid purified from antisera by immunoaffinity chromatography using antigen coupled to agarose beads at a concentration or dilution range of 10–20 µg/mL. Hydroxylamine (1M), phosphate-buffered saline (PBS, 0.01 M sodium phosphate, 0.25 M NaCl, pH 7.4), block solution (50 mM Tris, 0.14 M NaCl, 1% BSA, pH 8), and wash solution (50 mM Tris, 0.14 M NaCl, 0.05% Tween 20, pH 8) were prepared in-house. Deionized water (DI water, 18.2 MΩ·cm^−1^) was from an Easypure II ultrapure water purification system.

### 2.2. Preparation of Raman-Coded Microbeads

Monodisperse poly (styrene-co-AA) (PS) and poly (4-tert-butylstyrene-co-AA) (P4tBS) beads were prepared following a two-stage dispersion polymerization method [41]. In the first stage, 6.0 g of styrene or 4-tert-butylstyrene monomer, 0.24 g of AIBN initiator, 0.27 g of PVP 360,000 stabilizer, 0.30 g of 70% Triton X-305 co-stabilizer, and 34 g of 95% ethanol were charged to a 250 mL three-neck flask. The mixture was bubbled by nitrogen at room temperature for 40 min. The flask was then merged into a preheated 70 °C oil bath and stirred mechanically at 100 rpm for 1 h. In the second stage, the preheated comonomer AA solution (2 wt% of AA to the feed monomer mixed with 16 g of 95% ethanol) was added to the reaction flask using a syringe. The reaction was continued for another 24 h. For purification, the synthesized microbeads were washed three times with 95% ethanol and four times with DI water to remove any reaction residuals. The washed microbeads were rescattered in DI water, and the detailed contents of solids were measured by gravity analysis.

The functional carboxylic group on the microbeads’ surfaces was used to conjugate IgG (paratope) by EDC chemistry. In a typical experiment, 100 µL of 1.9 wt% purified microbeads and 100 µL of EDC/NHS/PBS (20 mg of EDC and 30 mg of NHS in 1 mL of phosphate buffer saline at pH 7.4) were mixed for 15 min at room temperature, followed by the addition of 10 µg of model paratopes (e.g., donkey antirabbit IgG for PS microbeads and donkey antigoat IgG for P4tBS microbeads). The mixture solution was incubated at room temperature for 2 h with gentle rotation and then quenched by using 1 M hydroxylamine. The conjugated microbeads were washed with PBS buffer and then mixed with 1 mL block solution (50 mM Tris, 0.14 M NaCl, 1% BSA, pH 8) for 30 min at room temperature. Finally, the microbeads were washed four times using wash solution (50 mM Tris, 0.14 M NaCl, 0.05% Tween 20, pH 8), and the paratope-conjugated microbeads were redispersed in DI water.

### 2.3. Preparation of SERS-Coded Nanotags and SERS-Coded Reporters

AuNPs were synthesized by reduction of gold (III) chloride by sodium citrate [42]. Briefly, 50 mL of 10^−3^ M HAuCl_4_·3H_2_O was brought to a boil, followed by rapidly adding 1 wt% sodium citrate at a mixing molar ratio of 1:2 [39]. After continuously stirring at the boiling temperature, a reddish-gold colloidal solution was obtained.

The SERS-coded nanotags were prepared by the formation of different self-assembled monolayers (SAMs) of functional thiols on AuNP surfaces. Here, the solution of 4-MBA or 3-MPA (2.5 µL, 10^−3^ M) was added separately to 1 mL of the above AuNP suspension under vigorous stirring for 2 min at room temperature, and the mixture solution turned purple. The resulting SERS nanotags were washed three times by operating centrifugation and redispersing cycles, and finally redispersed in 100 µL DI water. Hereafter, two different sets of epitopes (rabbit antihuman IgG and goat antihuman IgG) were separately attached to the performed SERS-coded nanotags via ionic and hydrophobic interactions [43]. Then, 5 µg of epitopes in phosphate buffer were separately charged to these 100 µL SERS-coded nanotag suspensions under gentle rotation at room temperature for 2 h. Then, 100 µL of 10% BSA solution was added to the mixtures and rotated for another 30 min, followed by three-time washing using DI water. The final epitope coupled SERS-coded nanotags (aka. SERS-coded reporters) were then redispersed in 100 µL DI water and stored at 4 °C.

### 2.4. Multiplex Immunoassays

For spectroscopic analysis, 50 μL of the two paratope-conjugated Raman-coded microbead suspensions were separately mixed with their matched and unmatched epitope-coupled, SERS-coded reporters. The mixtures were incubated for 1 h under gentle rotation at room temperature. After washing several times using DI water, the resulting microbeads were redispersed in 50 µL DI water. The air-dried microbeads on a glass substrate were subjected to Raman spectroscopic analysis. For multiplex immunoassays, a 50 μL mixture of two paratope-conjugated, Raman-coded microbeads were incubated with a mixture of two epitope-coupled, SERS-coded reporters for 1 h. After washing using DI water, air-dried microbeads on a glass substrate were subjected to Raman imaging analysis at characteristic wavenumbers of Raman microbeads and SERS nanotags.

### 2.5. Equipment

The images of microbeads and AuNPs were obtained by using a Quanta 450 scanning electron microscope (SEM) and a Phillips CM200 transmission electron microscope (TEM). The UV–vis absorption spectra were recorded using a Shimadzu UV-1601 UV–vis spectrophotometer. A LabRAM Horiba Raman microscope equipped with LabSpec 6 software was used to measure the Raman spectra and image microbeads, SERS reporters, and immunocomplexes. A 785 nm Xtra II diode laser from Toptica was applied for Raman imaging, with the monochromator comprising 600 grooves per mm grating. The Raman spectra were recorded using an acquisition time of 5 s and an accumulation time of 3 s. The SWIFT mode in LabSpec 6 was used for Raman imaging with an acquisition time of 1 s and a step size of 0.1 micron. The peak and CLS mode in the Raman microscope were used to generate the Raman false-color images to establish selective binding in the immunoassays. The CLS fitting was used to set up and perform the multivariate classical least squares fitting procedure on single spectra and multidimensional spectral arrays using a set of reference component spectra. This method is a supervised multivariate decomposition technique [44].

## 3. Results and Discussion

### 3.1. Raman-Coded Microbead Supports

**Figure 2:**
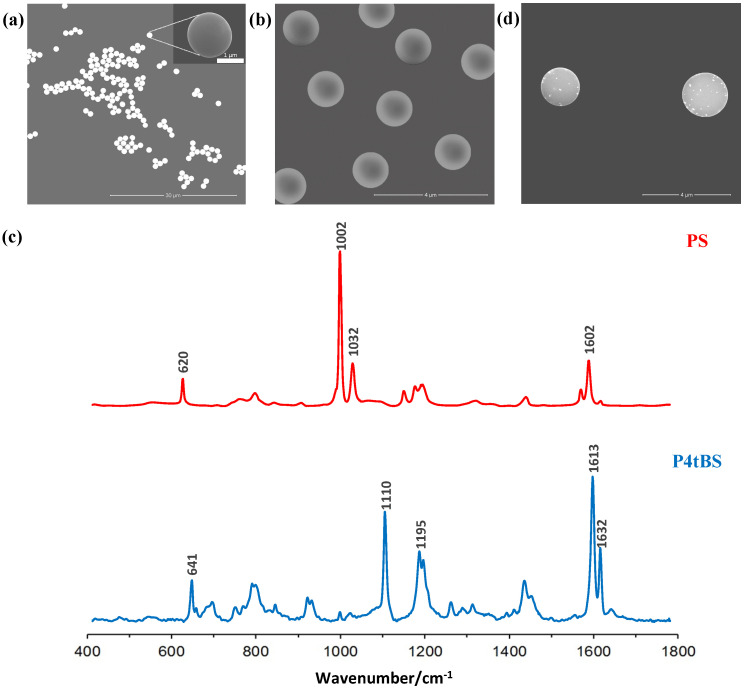
SEM images of monodisperse (**a**) PS and (**b**) P4tBS microbeads. (**c**) Raman spectra of these two Raman-coded microbeads with their characteristic wavenumbers labelled. (**d**) A typical SEM image of the immunocomplexes (SERS-coded reporters on the surface of Raman-coded microbeads formed through specific epitope-paratope interaction).

Raman-coded polymeric microbeads were used as the immune solid supports in bead-based immunoassays [45,46,47]. Dispersion polymerization was used for the synthesis of monodisperse polymer microbeads with average sizes of ~1.5 µm (Figure 2a,b) and narrow size distributions (CV < 1%), where a small amount of AA was introduced as the comonomer to generate functional carboxylic acid groups on the surfaces of microbeads [41]. These surface functional groups not only promoted microbead stability, but also allowed further surface paratope IgG immobilization via the EDC chemistry.

Styrene and 4-tert-butylstyrene monomers have different Raman spectra. Since polymers are long-chain molecules composed of many repeating monomers connected by covalent bonds, the Raman signals of their polymer microbeads are strong and distinguishable, which render them suitable as a Raman-coded support for bioanalysis. As shown in Figure 2c, the Raman spectrum of the PS microbeads showed a distinct vibrational band centered at 1002 cm^−1^ (strong), which was assigned to the υ1 symmetrical ring. Another peak related to the υ18A vibration was located at a wavenumber of 1032 cm^−1^ (medium) [48]. Similarly, the Raman spectrum of the P4tBS microbeads showed distinct vibrational peaks located at 1109 cm^−1^ (strong) and 1611 cm^−1^ (very strong). Due to small amount of surface carboxyl groups, no Raman spectral representation for carboxyl groups before and after EDC conjugation was evident.

### 3.2. SERS-Coded Nanotags and Reporters

**Figure 3:**
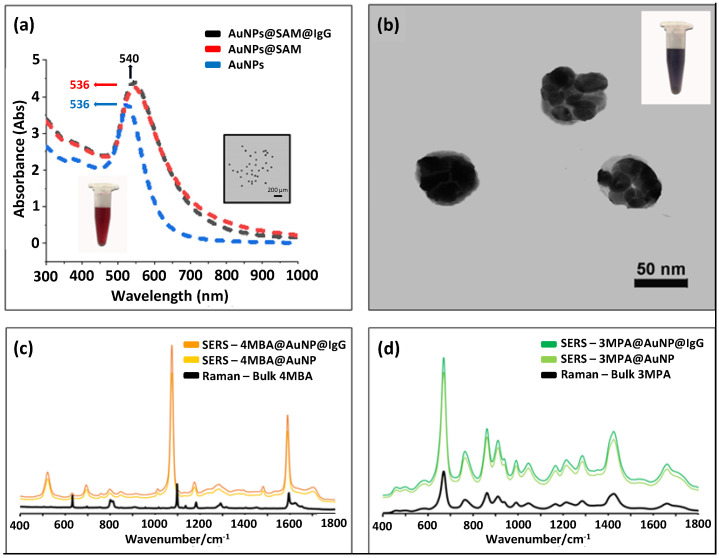
(**a**) UV–vis absorption spectra of AuNPs, AuNP@SAM, and AuNP@SAM@IgG; inset shows a TEM image of the AuNPs. (**b**) TEM image of SERS-coded nanotags. (**c**,**d**) Raman spectra of bulk 4 MBA/3 MPA, SERS-coded nanotags before and after epitope IgG loading.

The details of preparing the SERS-coded reporters are illustrated in Figure 1; SERS-coded nanotags were used to report the specific paratope–epitope interaction in the immunoassay analysis. The AuNPs used to prepare SERS-coded nanotags were synthesized at a 1:2 feed molar ratio of HAuCl_4_·3H_2_O and Na_3_Ct to achieve sufficient surface citrate ions and good colloidal stability. The localized surface plasmon resonance (LSPR) of the ~25 nm AuNPs showed a characteristic absorption peak at 525 nm (Figure 3a). The same amount of SERS-active molecules of 4-MBA and 3-MPA were separately mixed with AuNPs to produce the SERS-coded nanotags; the thiol groups of 4-MBA and 3-MPA had a strong affinity to the AuNPs, and carboxylic acid groups enhanced colloid stability [49]. The 4-MBA and 3-MPA formed SAM on the AuNPs via the Au–S bond, and an inevitable aggregation took place immediately after the SAM formation due to hydrophobic interaction [50]. The aggregates formed “hotspots” and facilitated strong SERS signals. The successful formation of SAM can be signified by an absorption-peak redshift to 536 nm upon addition of 4-MBA and 3-MPA [51]. The TEM image could be further used to identify the formation of SAM on the AuNPs and hotspots (Figure 3b); each aggregate contained several small AuNPs. Model epitopes of rabbit antihuman IgG and goat antihuman IgG were then mixed with the 4-MBA or 3-MPA SERS-coded nanotags separately to form two distinct SERS-coded reporters, and 10% BSA was used as a blocking solution to avoid future nonspecific bounding in bioanalysis. No redshift in the LSPR of the SERS-coded nanotags after epitope coupling indicated their core–shell–corona structure, in which the hydrophilic corona layer further enhanced the stability of the SERS-coded nanotags in aqueous solution.

Figure 3c shows the Raman spectra of bulk 4-MBA and the SERS spectra of 4-MBA on the AuNPs’ surfaces before and after epitope introduction. Both the Raman and SERS (with and without IgG) spectra revealed the characteristic vibrational peaks of the υ(CC) ring-breathing at 1074 and 1583 cm^−1^. However, other less intense peaks at the δ(CH) (1132 and 1173 cm^−1^) and υs(COO-) (1375 cm^−1^) were observed in the SERS spectra, but not the Raman spectrum of bulk 4-MBA [52,53,54]. Similarly, the Raman spectra of bulk 3-MPA, as well as the SERS spectra of 3-MPA on the AuNPs surfaces with and without IgG, are shown in Figure 3d. All spectra were dominated by three distinct peaks at 674, 867, and 1430 cm^−1^, which were assigned to the υ(CS)G, υ(SH), and υ(CH_2_) characteristic vibrational modes [55]. We also observed that the SERS signals before and after IgG (epitope) coupling were identical (Figure 3c,d), which agreed with the LSPR results, indicating no direct interaction between the AuNPs and IgGs.

The most intensive peaks of 4-MBA and 3-MPA were distinguishable with narrow bandwidths, resulting in their SERS-coded nanotags and reporters having different SERS signatures. Moreover, as the Raman scattering cross-sections of nanotags were much larger than bulk small molecules (4-MBA and 3-MPA), the intensities of Raman signals enhanced by AuNPs could reach a level of 10^6^. Such a great increase in the signal-to-noise ratio was favorable for highly sensitive analysis. The formed self-assembled gold colloid helped enhance the Raman signals of the 4-MBA and 3-MPA. Due to this enhancement effect of the SERS-coded nanotags, it was possible to lower the concentration limit for analyte detection. The increase in the intensity of SERS signal to Raman signal was quantified by the apparent effective enhancement factor (*EEF*) using the following expression:

$$EEF=\frac{I_{SERS}N_{bulk}}{I_{bulk}N_{SERS}}$$

where *I_SERS_* and *I_bulk_* are the intensities of the same band for the SERS and bulk Raman spectra, while *N_bulk_* and *N_SERS_* are the number of molecules for the bulk and SERS sample, respectively [56,57]. Under the experimental conditions, 2.5 μL 10^−3^ M of 4-MBA or 3-MPA was charged separately to 1 mL of 10^−3^ M AuNP aqueous solution to prepare the SAMs. Using the band of 1074 cm^−1^ for 4-MBA, the apparent EEF was calculated to be 1.08 × 10^6^. Similarly, using the band of 674 cm^−1^ for 3-MPA, the EEF was calculated to be 1.25 × 10^7^.

### 3.3. Immunocomplexes and Multiplex Immunoassays

We aimed to develop a novel bioassay for multiplex detection using Raman-coded microbeads and SERS-coded reporters; details are shown in Figure 1. In this study, donkey antirabbit IgG and donkey antigoat IgG were considered as model paratopes, while rabbit antihuman IgG and goat antihuman IgG were considered as model epitopes. The PS and P4tBS microbeads were separately immobilized with donkey antirabbit and donkey antigoat IgGs. On the other hand, rabbit antihuman IgG was coupled to the 4-MBA-coded SERS nanotag as the epitope of the donkey antirabbit paratope, and the 3-MPA-encoded SERS nanotag was decorated with the goat antihuman IgG as the epitope of the donkey antigoat paratope. Due to the specific recognition between these paired paratopes and epitopes, the SERS-coded nanotags were used to report the matched immune-interaction on the microbeads’ surfaces. In other words, in the presence of matched paratope–epitope pairs, both codes of the Raman bands from the microbeads and the SERS signals from the nanotags could be read simultaneously due to the formation of immunocomplexes. Otherwise, only the Raman bands of microbeads could be read in the presence of unmatched paratope–epitope pairs.

**Figure 4:**
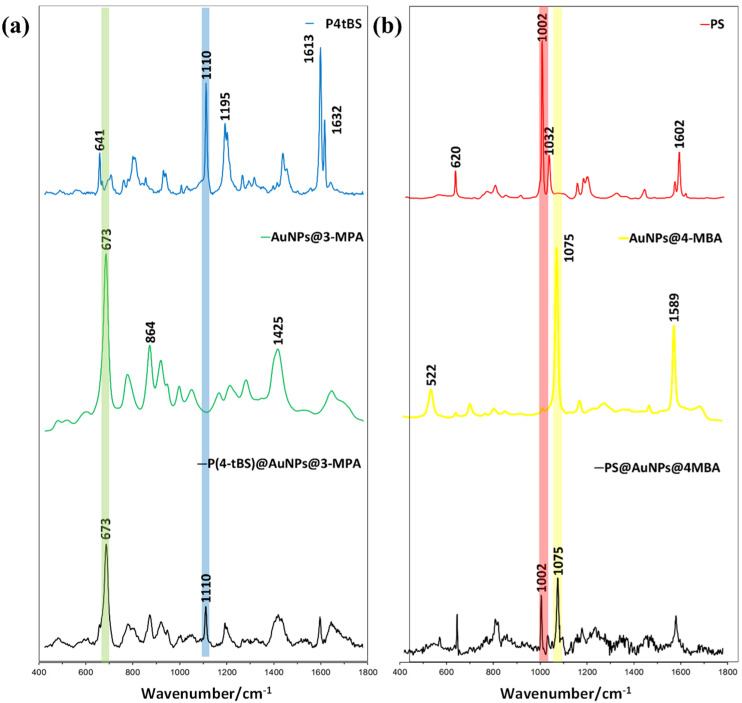
(**a**) Raman spectra of paratope-conjugated P4tBS microbeads, epitope-coupled AuNP@3-MPA SERS-nanotags, and the resulting immunocomplexes after the specific paratope and epitope interactions. (**b**) Raman spectra of paratope-conjugated PS microbeads, epitope-coupled AuNPs@4-MBA SERS-nanotags, and resulting immunocomplexes after the specific paratope and epitope interactions.

After mixing paired paratope-conjugated, Raman-coded microbeads and epitope-coupled, SERS-coded nanotags, the SEM image of the immunocomplexes in Figure 2d clearly showed the presence of SERS-coded reporters on the Raman-coded microbeads. To further conclude the specific biorecognition of the paratope-conjugated Raman microbeads to the counterpart epitope loaded to SERS-coded nanotags, we performed a Raman spectrum analysis (Figure 4a,b). As mentioned previously, PS microbeads displayed two strong Raman vibrational bands at 1002 and 1032 cm^−1^, whereas the SERS vibrational bands for 4-MBA were located at 1074 and 1583 cm^−1^. After the specific binding of the matched paratope and epitope, the individual Raman signals of the PS microbeads and the SERS signals of 4-MBA could be detected in the dry bead sample. Similarly, the P4tBS microbeads showed typical Raman strong vibrational bands at 1110 and 1613 cm^−1^, whereas the SERS vibrational bands of 4-MPA were located at 674 and 2576 cm^−1^. Again, due to the specific binding of the matched paratope and epitope, both the Raman signals of the P4tBS microbeads and the SERS signals of 3-MPA could be observed for the resulting dry microbeads. In presence of unmatched paratope–epitope pairs, only the Raman signals of PS or P4tBS could be observed. These results demonstrated that SERS reporters could selectively bind to microbeads through the specific matched paratope–epitope interaction with high sensitivity and selectivity. Experimentally, Raman shifts were reproducible in repeated experiments for Raman-coded microbeads and SERS-coded nanotags, as well as the paratope-conjugated microbeads with the matched or unmatched SERS-coded reporters. However, the intensity ratios of Raman-coded microbeads and SERS-coded reporters varied due to the inhomogeneous distribution of the antibody on the microbeads’ surfaces and difficulty in controlling the amount of loaded SERS-coded nanotags.

**Figure 5:**
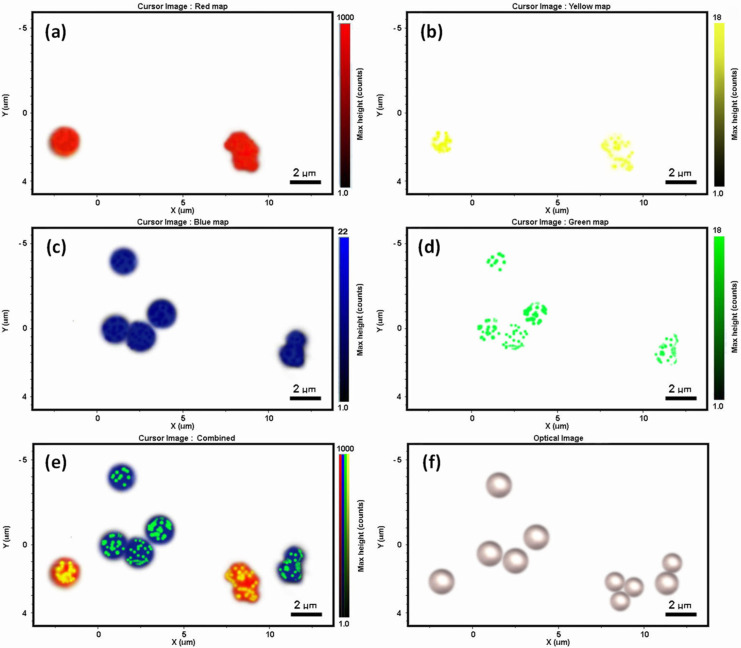
Raman imaging of the multiplex assays. (**a**–**d**) Raman images of PS microbeads (red) and 4-MBA SERS-reporters on PS microbead surface (yellow); P4tBS microbeads (blue) and 3-MPA SERS reporters on P4tBS microbead surface (green). (**e**,**f**) Combined false-colored spectroscopic and optical images of dry PS and P4tBS microbeads with their specific SERS reporters from immunoassays.

The specific recognition of paratopes and epitopes in the above immunoassays could be further examined by Raman mapping to explore future multiplex analysis through spectroscopic imaging of SERS nanotags on the Raman microbead surfaces. After mixing the two SERS-coded reporters with the two epitope-conjugated Raman-coded microbeads, the Raman images clearly showed the selective and specific interaction of paratopes and epitopes from one sample measurement. Figure 5f shows the optical image of dry microbead mixtures from this immunoassay, and no immunoprecipitation or aggregation was observed. That was important for the bead-based multiplex analysis to ensure high reproducibility and reliability. Raman mapping of this area was then carried out with a scanning step size of 0.1 micron. The false-colored Raman images were achieved based on the distinct spectral peaks of the Raman and SERS codes, as well as by using the classical least squares (CLS) algorithm mode. By selecting and deselecting distinct Raman signatures of the two polymer microbeads and the two SERS nanotags, different Raman mapping images were acquired (Figure 5a–d). From that, we could distinguish the microbeads (and their relevant paratopes) with or without SERS nanotags (and their relevant epitopes) (Figure 5e) simultaneously. Raman imaging revealed that there was no cross-interaction of the SERS nanotag signals on the microbead surfaces due to the presence of specific paratope and epitope interactions. Therefore, the false-colored Raman imaging analysis reinforced the Raman and SERS immunoassay results, and showed that the interaction was highly selective and highly specific.

The unabridged Raman spectrum and Raman imaging analysis indicated the specific recognition between matched paratopes and epitopes with high selectivity. This study combined the signatures of Raman-coded microbeads (support) and SERS-coded nanotags (reporter) for multiplex analysis. Since a variety of Raman-coded microbeads and SERS nanotags can be easily prepared, this Raman and SERS bioassay using vibrational information of microbead supports and SERS reporters can be expanded to simultaneous multiplex analysis from one homogeneous immunoassay. The capacity of the Raman dual-encoding system will be far more than that of a fluorescent coding system [58], qualifying its high analyte throughput.

## 4. Conclusions

A novel bioanalytical technique was demonstrated for multiplex analyte detection based on a Raman and SERS suspension immunoassay. Surface-functionalized, Raman-coded microbeads could be used as the support to immobilize various paratopes for bead-based immunoassays. AuNPs, SERS-active molecules, and epitopes could be used to prepare the core–shell–corona structured SERS-coded reporters. In a homogeneous immunoassay of mixed microbeads and SERS reporters, both Raman spectroscopic and Raman imaging analysis demonstrated that the SERS (from reporter) and Raman (from microbead) signatures could only be successfully read in the presence of specific paratope–epitope interactions. Such a system offered high selectivity, high specificity, high multiplex, no photobleaching, narrow spectra, and a single excitation wavelength when compared to a traditional bead-based fluorescent bioanalysis. This Raman and SERS immunoassay has great potential for future high-throughput multiplexing analysis of many target analytes from a single sample measurement.
